# Nursing intervention versus usual care to improve delirium among home-dwelling older adults receiving homecare after hospitalization: feasibility and acceptability of a Randomized Controlled Trail

**DOI:** 10.1186/s12912-016-0140-z

**Published:** 2016-03-14

**Authors:** Henk Verloo, Céline Goulet, Diane Morin, Armin von Gunten

**Affiliations:** University of Applied Nursing Sciences, La Source, 30, Avenue Vinet, CH-1004 Lausanne, Switzerland; Faculty of Nursing Science, University of Montreal, Montreal, Canada; Institut Universitaire de Formation et Recherche en Soins (IUFRS), Faculty of Biology and Medicine, University of Lausanne, Lausanne University Hospital, 10, Rte de la Corniche, CH-1010 Lausanne, Switzerland; Faculty of Nursing Science, Université Laval, Québec, Canada; Department of Psychiatry, Service Universitaire de Psychiatrie de l’Age Avancé (SUPAA), Lausanne University Hospital, CH-1008 Prilly, Switzerland

**Keywords:** Acceptability, Delirium, Feasibility, Intervention, Home-dwelling older adults, Prevention

## Abstract

**Background:**

Delirium is an acute cognitive impairment among older hospitalized patients. It can persist until discharge and for months after that. Despite proof that evidence-based nursing interventions are effective in preventing delirium in acute hospitals, interventions among home-dwelling older patients is lacking. The aim was to assess feasibility and acceptability of a nursing intervention designed to detect and reduce delirium in older adults after discharge from hospital.

**Methods:**

Randomized clinical pilot trial with a before/after design was used. One hundred and three older adults were recruited in a home healthcare service in French-speaking Switzerland and randomized into an experimental group (EG, *n* = 51) and a control group (CG, *n* = 52). The CG received usual homecare. The EG received usual homecare plus five additional nursing interventions at 48 and 72 h and at 7, 14 and 21 days after discharge. These interventions were tailored for detecting and reducing delirium and were conducted by a geriatric clinical nurse (GCN). All patients were monitored at the start of the study (M1) and throughout the month for symptoms of delirium (M2). This was documented in patients’ records after usual homecare using the Confusion Assessment Method (CAM). At one month (M2), symptoms of delirium were measured using the CAM, cognitive status was measured using the Mini-Mental State Examination (MMSE), and functional status was measured using Katz and Lawton Index of activities of daily living (ADL/IADL). At the end of the study, participants in the EG and homecare nurses were interviewed about the acceptability of the nursing interventions and the study itself.

**Results:**

Feasibility and acceptability indicators reported excellent results. Recruitment, retention, randomization, and other procedures were efficient, although some potentially issues were identified. Participants and nurses considered organizational procedures, data collection, intervention content, the dose-effect of the interventions, and methodology all to be feasible. Duration, patient adherence and fidelity were judged acceptable. Nurses, participants and informal caregivers were satisfied with the relevance and safety of the interventions.

**Conclusions:**

Nursing interventions to detect/improve delirium at home are feasible and acceptable. These results confirm that developing a large-scale randomized controlled trial would be appropriate.

**Trial regestration:**

ISRCTN registry no: 16103589 - 19 February 2016

## Background

Delirium is a clinical syndrome characterized by a mental disorder involving acute onset and disturbances to consciousness, orientation, memory, thought, perception, and behavior that tend to fluctuate during the course of the day [1]. The syndrome’s etiology is often multifactorial, with high occurrence rates—ranging up to 83 %—among hospitalized older patients [1, 2]. One third of these confused older patients still have unresolved delirium symptoms when they return home [3].

The prevalence of delirium among home-dwelling older adults is estimated to lie between 0.50 % and 34 %, but well-documented population-based data is still lacking [3, 4]. Delirium can be a reversible syndrome if detected early and, in many cases, prompt detection of risk factors have been helpful in successfully avoiding it [5]. Delirium is considered to be a reversible condition if detected early, and literature indicates that prompt detection of delirium risk factors can avoid its occurrence in many cases [5]. Undetected and untreated delirium can have serious consequences for older adults such as cognitive and physical decline, re-hospitalization, institutionalization or premature mortality [6, 7]. A preventive approach to delirium, including the detection of risk factors by community health nurses, may contribute to significantly maintain or restore health among vulnerable older adults [8]. Delirium detection and intervention strategies are frequently applied to hospitalized older patients [1]. However, strategies targeting delirium in home-dwelling older adults (and their informal caregivers) are largely unexplored [9, 10]. Previous studies have suggested that multicomponent interventions for preventing delirium are related to better patient outcomes [11]. Although poorly documented to date, it is nevertheless reasonable to suggest that rapid detection and preventive interventions by community health nurses might significantly contribute to maintaining or restoring the health of vulnerable older adults at a high risk of developing delirium [8]. Indeed, to the best of our knowledge, patient-centered research based on homecare nursing interventions designed to detect and improve delirium in home-dwelling older patients after hospital discharge is inexistent [12].

This pilot trial aimed to assess the feasibility and acceptability of a new nursing evidence-based intervention developed to detect and improve delirium among home-dwelling older adults, including their informal caregivers. The research questions were: 1) Are the methods for assessing symptoms/signs of delirium and the overall study procedure feasible for use with home-dwelling older adults? 2) Are the interventions and study procedures acceptable for discharged, home-dwelling older adults, their informal caregivers, and homecare nurses?

## Methods

### Study design

A pilot randomized controlled trial was designed and conducted to assess how hospital-discharged home-dwelling older adults, their informal caregivers, and their homecare nurses rated the feasibility and acceptability of an innovative nursing intervention and the study procedures themselves.

### Intervention development

The present study’s nursing intervention was developed based on: a) the United Kingdom’s Medical Research Council guidelines [13]; b) the theoretical framework of stressor prevention strategies in the Neuman Nursing Model [14]; c) the intervention mapping approach developed by Bartholomew et al. [15]; and d) guidelines from several professional organizations and recently published geriatric-friendly hospital programs [13, 15–24].

Participants in the control group (CG) received the usual comprehensive care provided by homecare nurses in French-speaking Switzerland. The precise nature of that care depended on the patient’s clinical status, the presence of informal caregivers, and the range of skills of the nursing staff at the homecare center.

In addition to the usual care dispensed by homecare nurses, participants in the experimental group (EG) underwent five patient-centered nursing interventions provided by a gerontology clinical nurse (GCN) during five home visits. Interventions were separated into seven domains: assessment, detection, monitoring, support, direct care, health promotion, and education. These domains contained 15 nursing-specific protocols, covering 70 nursing activities (Table 1) previously judged to be acceptable for patients by a panel of community nursing experts. Furthermore, the nursing intervention had previously been adapted and standardized following pre-testing with five discharged, home-dwelling older patients. A user’s guide was developed in order to structure and standardize the intervention. This work, prior to the pilot study, allowed the researchers to select the most appropriate, patient-centered domains, protocols, and activities for older adults according to their clinical needs, including in presence of delirium symptoms and risk factors. This type of approach is often referred to as a tailored approach because it makes the link between standardized guidelines and patient-centered care [16].

**Table 1:** Intervention to detect and prevent delirium among discharged home-dwelling older adults after hospitalization or acute illness

Phase	Timing (Estimated duration)	Domain	Description of activity
Phase I	ASSESSMENT OF CLINICAL STATE AND DELIRIUM RISK FACTORS		
	Start of the intervention (15 mins)	Assessment	• Symptoms/signs of delirium • ADL/IADL performance • Biological parameters • Comprehension among informal caregivers
		Detection	• Prodromal symptoms • New delirium risk factors
Phase II	PATIENT-CENTERED INTERVENTION		
	Subsequent to assessment of clinical state and delirium risk factors (30 to 40 mins)	Monitoring	• Cognitive impairment • Sensorial impairment • Constipation/diarrhea • Obesity/sarcopenia • Infection • Polymedication • Sleep-wake cycle • Mobility impairment, fall risk • Pain • Debilitating comorbidities
		Care	• Verify support for ADL/IADL • Verify nursing care activities such as catheter care, wound-dressing, medication preparation • Verify effectiveness of pain management • Verify care needed by informal caregivers
		Support	• Physical • Cognitive • Psychological and emotional • Spiritual • Organized support for informal caregivers
		Education	• Delirium • Healthy aging • Prevention of skin, urinary, and pulmonary infections • Fall prevention • Adherence to medication therapy • Prevention of excessive alcohol consumption and use of over the counter medication • Therapeutic education on healthy aging to informal caregivers
	RECOMMENDATIONS		
	End of intervention (5 mins)	Individualized healthy aging strategies	• Preventing physical discomfort • Mobility, nutrition, and hydration strategies • Cognitive stimulation strategies

### Patient-centered nursing intervention

GCNs started each first patient visit with an assessment of their symptoms/signs of delirium using the Confusion Assessment Method (CAM), of their level of autonomy using the Index of activities of daily living (ADL/IADL), and of their biological parameters (glycemia, blood pressure, heart rate, self-evaluation of pain, oxygen saturation, and body temperature). In the presence of symptoms/signs of delirium on the first visit or of worsening patient conditions on subsequent visits, the GCN informed and reassured the older adult and his informal caregiver. The GCNs used an empathic approach to educating them about the symptoms/signs of delirium, passing on educational material, and to advising them, when judged necessary, to contact their family physician. This first intervention was made within two days of participants giving their consent.

In the second phase, during subsequent visits, GCN provided tailored interventions and nursing activities—based on detection, care needs, health education and health promotion—according to the presence of delirium or potential delirium risk factors (Table 1). These interventions—numbers two to five—were conducted at 3, 7, 14, and 21 days after their consent. These intervals were chosen based on the average duration of episodes of delirium; these usually vary between three to seven days [1]. Table 1 presents the patient-centered nursing intervention strategy.

### Sample and setting

This pilot study was conducted from February to November 2012 in collaboration with a home healthcare unit in French-speaking Switzerland. Older patients with a medical prescription for homecare were eligible to participate if they: i) were aged 65 years old or over; ii) had been recently discharged from hospital; and iii) were capable of understanding and answering questions in French. They were excluded if they: i) received out-patient treatment in a hospital on a regular basis; ii) already had a medical prescription for a single intervention of homecare; and iii) lived outside the study’s geographical limits. The participants were enrolled and assessed within 48 h of hospital discharge with the aim of rapidly detecting the symptoms/signs of delirium or delirium risk factors.

### Sample size and randomization

One hundred and fourteen participants were recruited to the study and assigned to groups using computerized block randomization (4 participants per block) using opaque sealed envelopes. They were allocated to either the EG (*n* = 56) or the CG (*n* = 58). Totals of 51 EG participants and 52 CG participants completed the study. Every participant, the principal investigator (PI), and the GCNs who carried out the interventions were aware of the group allocations because nursing interventions needed to be prepared and adapted to the patients. As a pilot study, it was not necessary to calculate sample sizes [25], however, this study managed to recruit a sample almost equal in size to the few other existing studies on delirium prevention in hospital settings [26, 27].

### Instruments and procedures

The PI and GCNs collected data on the socio-demographic characteristics, symptoms/signs of delirium (CAM), delirium risk factors, cognition (MMSE), physical status (ADL/IADL), comorbidities (Cumulative Illness Rating Scale for Geriatrics, CIRS-G), and medication use of both groups. These data were collected through questionnaires, patient records, and face-to-face interviews. Inter-rater reliability between the PI and the GCNs was excellent with regards to the assessment of the CAM and ADL/IADL (with Cohen’s Kappa coefficients of 0.79 and 0.85, respectively), and the results of the MMSE and CIRS-G (with intraclass correlations of 0.92 and 0.83, respectively) [28]. The PI and GCNs also collected data on the duration of the baseline assessment (mins/assessment), the number of interventions (1–5), the duration of these interventions (mins/intervention), the duration of the assessments of symptoms/signs of delirium using the CAM in patient records, and using measurement instruments (mins/assessment) at study entry (M1) and after one month (M2). Besides duration, data related to interventional and methodological feasibility and acceptability were also collected throughout the study; these came from the personal notes written by the PI and the CGNs, as well as the comments from participants and informal caregivers; these were analyzed subsequently [29, 30].

More specifically, acceptability data were collected about recruitment, the participant retention rate, and the number and timing of nursing interventions. Additionally, the GCNs assessed their own adherence to the number of patient-centered nursing interventions and their completion rate for the full set of interventions. At the end of the study (M2), older adults and their informal caregivers from the EG answered open-ended questions on the acceptability and safety of nursing activities. With regards to feasibility and acceptability, each intervention was examined using the number of procedures carried out as well as the barriers and facilitators to of the procedures in data collection and the organization of the strategy (Table 2).

**Table 2:** Evaluation of feasibility and suitability among home-dwelling older adults and their informal caregivers

Feasibility	- *Recruitment* - *Retention rate* - *Adherence to the interventions* - *Duration of the interventions* - *Suitability and safety of the interventions* - *Difficulties and constraints during interventions*
Suitability	- *Duration of measurement* - *Adequacy of the recruitment strategy* - *Adequacy of the randomization process* - *Adequacy of the data collection process* o *Accessibility of data in a home healthcare setting* o *Data collection from participants and their informal caregivers* - *Adherence and fidelity to the study procedures* - *Adequacy of the intervention procedures* - *Adequacy of the intervention content* - *Adequacy of the number of nurse interventions* - *Facilitators and barriers to intervention fidelity*

### Data analysis

Comparisons within and between groups were performed, and the most appropriate statistical tests were selected according to the type of variables. Statistical analyses were performed using the IBM-Statistical Package for Social Sciences (IBM-SPSS®), version 21 [31]. A statistical significance threshold value was established at *p* = 0.05, and all tests were two-tailed. Qualitative data were summarized using content analysis [32, 33]. In order to assess the number of symptoms/signs of delirium using the CAM, as documented in patient records after the provision of usual care by community health nurses, well-established analytical approaches were used [34, 35].

## Results

### Socio-demographic profiles

The two groups did not differ significantly with regards to socio-demographic characteristics, health status, or risk factors of delirium. The majority of participants were widowed older women (>82 years old), living alone, and with a lower or vocational level of education. Loss of independence after an acute illness was the most common reason for being prescribed homecare services. The health status of participants, as measured using the MMSE, CAM and Geriatric Depression scale (GDS), showed important rates of comorbidities, with high average numbers of symptoms and signs of cognitive impairment, delirium, and depression; daily polymedication often included more than six drugs. No significant difference was observed between the mean number of usual care interventions provided by community healthcare nurses in the EG and the CG. More than half of the participants indicated being stressed by situations involving personal conflict (Table 3). Furthermore, participants did not differ significantly from those who refused to participate.

**Table 3:** Basic assessment of participants’ socio-demographic characteristics, health status, and delirium risk factors

Variables	Experimental group (*n* = 51)	Control group (*n* = 52)	*P*
Age (years)			
Average (*SD*)	82.92 (*6.73*)	83.50 (*7.62*)	.249^a^
Gender			
Female (%)	33 (64.6 %)	34 (65.4 %)	.942^b^
Civil status			
Single	3	3	
Married/partner	21	18	.664^c^
Divorced/separated	4	2	
Widowed	23	29	
Living with			
Partner/spouse	23	15	.624^c^
Close family member	6	4	
Education			
Primary	3	10	
Secondary	20	18	.158^b^
Professional	19	13	
University	9	11	
Reason for home healthcare			
Accident	13 (25.5 %)	14 (26.9 %)	
Illness	38 (74.5 %)	36 (69.2 %)	
Respite care informal caregivers	0 (0 %)	2 (3.8 %)	.353^a^
Usual number of weekly homecare visits			
Min–Max	1–7	1–7	
Average (SD)	2.26 (1.34)	2.28 (0.84)	.916^a^
Health status – comorbidities			
Symptoms of delirium (average no.)	2.71	2.38	.395^c^
MMSE (average score)	23.96	23.81	.873^a^
IQCODE	3.69	3.67	.895^a^
Functional Status (ADL/IADL)	32.16	32.02	.938^a^
CIRS-G	13.45	14.04	.354^a^
Depression (GDS-30)	9.10	8.32	.432^c^
Nutritional status (BMI)	23.62	23.26	.678^a^
Pain assessment (EVA)	2.73	3.37	.367^c^
Pharmacological delirium risk factors			
Average # medication (*SD*)	6.22 (2.87)	6.42 (2.69)	.706^a^
Delirium high risk # medication^d^ *(SD)*	1.16 (*1.20*)	1.06 (*1.03*)	.655^a^
Delirium medium risk # medication^d^ *(SD)*	0.71 (*0.67*)	0.69 (*0.85*)	.929^a^
Delirium uncertain risk # medication^d^ *(SD)*	4.35 (*2.37*)	4.63 (*2.29*)	.541^a^
Non-pharmacological delirium risk factors			
Urinary in-dwelling catheter/wound	16 (31.4 %)	18 (34.6 %)	.726^e^
Conflict with partner/spouse	29 (56.9 %)	25 (48.1 %)	.372^e^

*Note*. ^a^Student *t* test; ^b^Fisher Exact Test; ^c^Mann-Whitney *U* test; ^d^following the American Geriatrics Society 2012 Beers Criteria Update Expert Panel, (Resnick & Pascala, 2012); ^e^ Pearson’s Chi-square test

### Acceptability of the intervention

- *Recruitment*

During the recruitment period, 319 medical prescriptions and discharge reports requesting homecare for older adults were reviewed in order to determine their eligibility. Of 196 older adults eligible for the study, 58 % (113/196) agreed to participate. Of the 82 refusals, 25 participants were not interested in the study, 12 declared receiving too many interventions as it was, 10 declared themselves to be too tired to participate, six refused home healthcare, two were immediately re-hospitalized, and one was immediately admitted to a nursing home. Furthermore, six family members opposed the participation of their relative.

- *Retention rate*

The study’s 91 % (103/113) participant retention rate was judged very high. Retention rates between the EG and the CG were very similar (91 % or 51/56, and 90 % or 52/58, respectively). Four participants in the EG abandoned the study because one died, one changed geographical location, one was institutionalized, and one gave no reason. Six participants in the CG abandoned the study because four were institutionalized, one died, and one left the study.

- *Adherence to the intervention program*

**Fig. 1:**
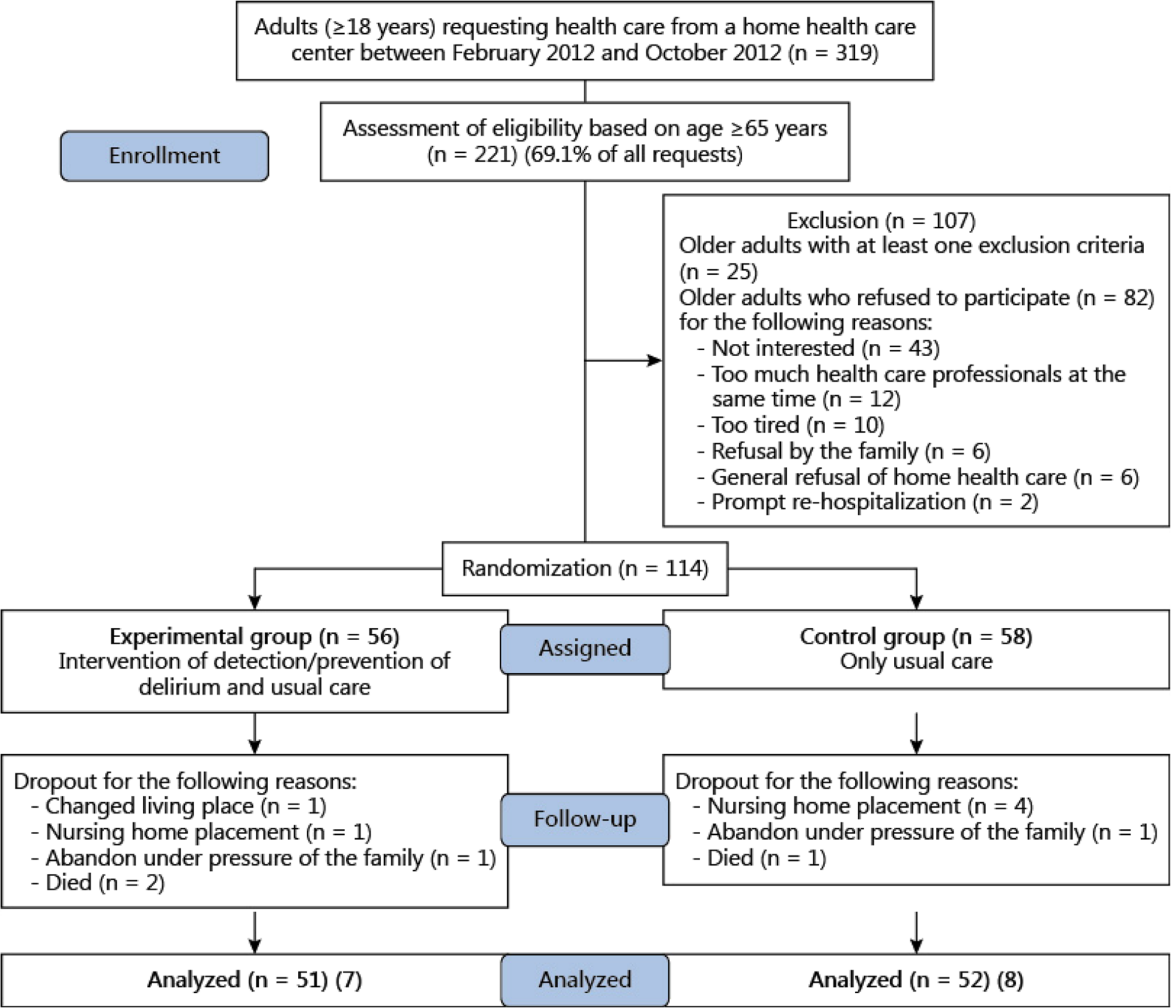
Recruitment of the participants

A total of 244 patient-centered nursing interventions were carried out for the 51 EG participants. Forty-four participants received all five nursing interventions, three received only four, and four received only three (Fig. 1). Family members were present during 104 interventions.

- *Intervention duration*

The duration of interventions varied between 5 and 180 mins; average duration was 59.8 mins. Details of the minimum, maximum, and average duration of interventions one to five are detailed in Table 4. Intervention one took an average of 12 mins more than intervention five. Using a posthoc Bonferroni correction of the *p-value* at 0.013, only the shorter minimum durations of interventions two and three showed a significant difference when compared to the average duration of intervention one (Table 4).

**Table 4:** Duration of patient-centered nursing interventions

Intervention	Duration^a^			95 % CI^b^		*p*-value
	Min^a^	Max^a^	M (SD)^a^	IL^a,c^	UL^a,d^	
Intervention 1	10	120	66.4 (25.7)	57.6	72.3	-
Intervention 2	10	120	61.4 (24.6)	55.3	70.1	0.004*
Intervention 3	5	180	59.1 (31.6)	50.9	69.8	0.002*
Intervention 4	5	120	55.5 (23.8)	49.4	64.0	0.013
Intervention 5	5	150	54.0 (27.3)	45.7	62.3	0.027

*Note:* ^a^ in mins; ^b^Confidence Interval; ^c^Inferior level; ^d^Upper Level; *Significant with *p*-value < 0,013 after Bonferonni correction

- *Acceptability of the nursing interventions at home*

The participants and their informal caregivers stated that all the interventions provided during the homecare visits (*n* = 244) had improved their quality of life and decreased the discomforts of their ill-health. All the participants collaborated actively during the interventions and expressed their satisfaction directly to the homecare nurses. Participants with a high-school level education or higher were particularly interested in health education and health promotion information concerning delirium risk factors.

The qualitative data was collected among 44 out of 51 (79 %) of the EG participants during the last nursing intervention. Open-ended questions were used in a semi-structured interview (before T1). These interviews were recorded and summarized using content analysis [32, 36]. They stated that the interventions they had experienced had been both efficient and patient-centered. Favorable progress in the health status of the three participants who had undergone an elective hip replacement meant that homecare visits stopped after intervention three, and a further four participants stopped after intervention four. Two supplementary explanations were necessary to clarify non-pharmacological risk factors of delirium and to ensure that patients adhered more closely to the prescribed medication doses. During the final intervention, seven participants stated that the number, content, duration, and intensity of the interventions provided had been well adapted to their health status.

- *Acceptability and safety of interventions*

Homecare nurses mentioned the positive feedback that they had received from EG participants; these indicated that nursing interventions had caused no safety issues, whether physical (falls) or psychological (increased anxiety). Nurses’ observations also highlighted the inappropriate accommodation conditions endured by some of the participants suffering from mobility and sensorial impairment. Neither the M1 and M2 assessments nor the EG interventions were in any way considered to be disruptions to the homecare nurses’ usual activities or to the homecare setting.

- *Difficulties during interventions*

The study recorded a number of complications to carrying out interventions: seven participants had difficulties reading and writing in French; 12 participants had a sensorial impairment and were unable to answer simple questions, which required rapid adaptations to the interventions. Less than 50 % of the informal caregivers were present during the consent procedure (*n* = 48), and even fewer were present during the interventions (*n* = 23), due to work commitments. In 13 % of interventions, existing or latent personal conflicts between participants and their caregiver or partner reduced the applicability of certain intervention components.

### Feasibility of interventions and the study as a whole

- *Duration of measurement and organization*

The time needed to fill in the forms on health status and delirium risk factors at M1 varied between 30 and 210 mins (M = 75 mins). At M2, the time required fell to between 23 and 150 mins (M = 39 mins). The significant difference found between the time needed at M1 and M2 (95 % CI [28.58, 43.07]) might be explained by the time needed to explain to participants and their caregivers how to fill in the questionnaires. The time needed to fill in the CAM form using clinical observations from patient records was also assessed. The time required to complete a CAM form (1,300 CAM forms from 103 patient records) varied from 15–35 mins, depending on the number of usual care interventions (minimum 1 to maximum 7 weekly homecare events) carried out during the study period.

During the study period, the PI was present eight hours a day, five days a week. This enabled the PI to regularly recruitment participants, develop good communication with the homecare nurses, and avoid overlapping appointments between home visits to the CG and EG.

- *Adequacy of the recruitment strategy*

Immediately after the prescription of nursing care in a homecare setting, eligible patients were contacted for a prompt appointment to explain the pilot study and the anticipated demands of participation, and to sign an informed consent form. Informal caregivers were also invited to be present. Both patients and informal caregivers were given 48 h to give consent. The recruitment period was spread over six months. Overall, 319 homecare prescriptions were reviewed, and 69 % were deemed eligible (221/319). Non-eligibility was mostly related to the prescription of a single homecare intervention, to the fact that patients were also receiving outpatient treatment in the hospital, or to the fact that they lived outside the study’s catchment area.

- *Adequacy of the randomization process*

Computerized block randomization using opaque sealed envelopes containing the group assignments was adequate for allocating comparable participants to the EG and CG (Table 3). This approach allowed the timing of the five nursing interventions in the EG to be coordinated and contributed to the feasibility of the intervention strategy. Four participants randomized to the CG asked to have their assignment changed to the EG, but no changes in the groups were permitted.

- *Adequacy of data collection*

- ◦ *Accessibility to homecare data*

Data on health behavior, health status, current medical treatments, the frequency of planned care, hospitalizations, institutionalization, and deaths were available through patients’ homecare records. The transcripts of the observational notes taken by the homecare nurses, and kept in patients’ records, revealed that they were incomplete in 77 % of cases, suggesting that several symptoms/signs of delirium remained undetected.

- ◦ *Data collection from participants and their informal caregivers*

In three instances at the time of intervention, participants were under the influence of alcohol and had difficulty filling in their questionnaires. Nine participants refused to draw a figure or write a sentence during the administration of the MMSE. Fifteen participants had no support from an informal caregiver so the Informant Questionnaire on Cognitive Decline in the Elderly (IQCODE) could not be completed. Difficulties in obtaining reliable answers on the GDS were also observed in participants who scored less than 15 points on the MMSE, and four of these were unable to even understand the instructions needed to fill in the GDS and the MMSE. Two interventions overlapped with usual care activities and six patients were absent for their M2 assessment.

- *Adherence to the study and the intervention procedures*

A well-organized timetable facilitated adherence to the study’s methodology. For example, eight participants presenting with important cognitive impairment needed the date of their M2 assessment adjusted, and the presence of their informal caregivers was requested. Despite the stress endured by some participants and their informal caregivers when it came to filling in the MMSE, the GDS and the IQCODE, the home health nurses mentioned that all the participants and their informal caregivers appreciated the follow-up contact at M1 and M2 and the time and interest taken to assess how they were experiencing life at those moments.

A strict schedule and attentive follow-up prevented absences and overlaps with private or regular care appointments. It was necessary to establish a maximum duration (2 h and 30 mins) for interventions with participants suffering from loneliness or social isolation so that homecare nurses did not overstay.

- *Adequacy of intervention content*

According to the majority of participants and their informal caregivers (44/51), interventions generated health benefits during the post-hospitalization period. They mentioned improved quality of life and general well-being, as well as some relief from discomforts such as pain, constipation, nutrition, dehydration, or lack of mobility. The relational, psychological, and health promotion support provided by the GCNs during their interventions corresponded to the emotional, psychological, and physical needs expressed by almost all the participants and their caregivers. Repeated explanations and reminders helped to ensure better adherence to prescribed doses of medication and to substantially reduce pharmacological risk factors for delirium.

- *Adequacy of number and spacing of interventions (nurse-dose)*

One hundred and six (43 %) interventions were made in the morning (8 h00 to 12 h00), and 138 (57 %) in the afternoon (13 h00 to 19 h00). No problems were observed during these interventions with regards to the intensity of the health promotion or health education activities or with regards to physical fatigue/exhaustion. Twenty-five percent of participants judged interventions one and two to be too close together, however.

- *Facilitators and barriers to adherence to methodology*

Some of the patients’ socio-demographic characteristics facilitated adherence to the intervention methodology. Nine participants principally communicated with their CGN in their Italian mother tongue, which was considered to be a factor facilitating adherence. Also, participants with higher levels of education were interested in the health education and health promotion aspects of interventions, particularly information related to the pharmacological risk factors of delirium.

One barrier to patient-centered nursing interventions with participants with impaired mobility was cluttered living spaces, including rugs rather than fitted carpets and inaccessible elevators. Also, participants with advanced cognitive and hearing impairments, or with multiple symptoms/signs of delirium required the presence of an informal caregiver or family member in order to receive individually adapted interventions. Three intoxicated participants significantly reduced the value of interventions on two visits.

## Discussion

Numerous conclusions can be drawn from this pilot study. We can confirm that the Neuman Systems Model is an appropriate framework to underpin prevention as a nursing intervention. This theoretical framework allows a clear and reliable identification of delirium risk factors and its early and efficient detection. It also fosters the development of a nursing prevention strategy at home [14].

Further, this study directly targeted home-dwelling older adults presenting symptoms of delirium or delirium risk factors. The content and intensity of the five additional patient-centered nursing interventions were adapted to the severity of the patients’ cognitive and functional decline and aimed to prevent risk factors for delirium. Six domains of activity were implemented during each intervention, starting with the assessment and detection of the symptoms of delirium and new risk factors, and the measurement of biological parameters. The favorable evolution of the cognitive and functional status of the EG participants demonstrated the adequacy, clinical appropriateness and relevance of the sequence of interventions. This study was well accepted by the participants, their informal caregivers, and the nurses involved. Thus, we believe that the components of this pilot study should be considered as implementable in any future experimental randomized clinical trials [37].

The difference in duration between intervention one and the other four was mainly due to the time spent reinforcing the nurse-patient relationship, and building confidence and empathy with participants and their informal caregivers. Time was also required to answer questions about how the four following interventions at home would be organized. Time also helped nurses to detect loneliness and social isolation, both of which are considered important risk factors for delirium [38]. Extra time spent during intervention one probably contributed to motivating participants to actively apply nursing recommendations, thus resulting in high levels of adherence.

The assessment of symptoms/signs of delirium using the CAM and patients’ clinical records seems feasible. However, an important amount of incomplete clinical patient records were observed. Several reasons can be mentioned. First, the daily practice of community health nurses is based on oral transmission and less on written documentation of the clinical symptoms of their patients. Written communication is mostly considered as time comsuming and should be considered as an important barrier in relation to the use of patient records to detect delirium symptoms [39]. Secondly, It is well documented that detection of delirium symptoms is a major issue for nurses with a risk of under-detection of delirium [40, 41]. Hare et al. and Malenfant et al. documented that nurses have inadequate levels of knowledge of delirium symptoms [42, 43]. This could result in under-documentation of the delirium symptoms by the community health nurses, and this corroborates with the results of Voyer et al., Morandi et al. and Steis et al. [35, 44, 45]. Future studies should explore innovative strategies for collecting daily health data from home-dwelling older adults with or at risk of symptoms/signs of delirium.

The successful recruitment of participants to the study and their rapid acceptance of it can be related to several elements. Firstly, the PI spent considerable time informing homecare nurses about the usefulness and relevance of a pilot study. This resulted in a clear commitment from them to encourage homecare patients to participate. This shows that homecare or community health nurses can play a key role in research by spreading information and encouraging eligible patients to participate in experimental studies [37]. Other factors facilitating the study’s organization and important to successful recruitment were the single center setting, the lack of concomitantly ongoing clinical studies, and a small catchment area including a high demographic density of patients.

Although recruitment was considered successful, analyzing the reasons for refusals to participate might contribute to optimizing future recruitment strategies in similar studies. Indeed, half of the discharged older adults eligible refused to participate, stating, for example, that participation would mean too many forms of care at the same time, or that they were too tired. The literature shows that these types of reasons have proven to be barriers to consent in other studies [46, 47]. Other reasons for refusing to participate were fears about institutionalization or the risk of losing one’s driver’s license [48]. Finally, families who refused to support the participation of an aged relative may have feared a loss of family privacy, the disclosure of family conflicts, or other personal problems.

This present study had some limitations with regards to the feasibility and acceptability of the interventions and the study as a whole. The most significant limitation in this study concerns the onset and the delay between the discharge of the older adult and the first assessment of delirium with the CAM within the 2 days after having been discharged home. Delirium could have been missed with dramatic consequences such as readmission to the hospital or early and avoidable mortality [49]. An important concern in this study was the trustworthiness of the collected qualitative data among cognitive impaired older adults [37]. The facts that newly discharged older adults are exhausted, and community health nurses are overworked or face serious time constraints, meant that a more structured interview and better-organized data registration were not feasible. Another limitation to this study is the limited feedback and collaboration from informal caregivers during intervention visits. However, we estimate it unlikely that these shortcomings can take much away from the acceptability of this innovative approach

## Conclusions

This pilot study demonstrated the beneficial effects of the patient-centered nursing interventions aiming to improve delirium at home [3]. The Neuman Systems Model theoretical framework revealed itself to be appropriate for conducting nursing intervention studies of delirium at home [50]. Participants, informal caregivers, and homecare nurses expressed their satisfaction throughout the different stages of the intervention strategy and the study as a whole; they also considered the interventions to be acceptable, and their number and spacing appeared to be feasible. These results are promising; they challenge researchers to develop similar projects with the ultimate aim of allowing older adults to remain at home longer in optimal conditions of physical and mental health.

### Ethics approval and consent to participate

The regional Research Ethics Committee of the Canton of Valais approved the study protocol (CCVEM 030/11). After presenting and explaining the study to potential participants, they were required to sign informed consent forms; if they scored below 15 points on the MMSE, then their informal caregivers were asked to sign instead.

## Abbreviations

ADL/IADL: Katz and Lawton Index of activities of daily living
CAM: confusion assessment method
CG: control group
CIRS-G: cumulative illness rating scale for geriatrics
EG: experimental group
GCN: geriatric clinical nurse
GDS: geriatric depression scale
IQCODE: informant questionnaire on cognitive decline in the elderly
M1: start of the study
M2: study after 1 month
MMSE: mini-mental state examination
PI: principal investigator
